# The New Rival of Cocaine

**Published:** 1887-10

**Authors:** 


### CULLINGS FROM CONTEMPORARIES.

THE NEW RIVAL OF COCAINE.—That "truth is stranger than fiction" the experiences of daily life frequently affirm. We live and move in the midst of facts which, when once revealed to our senses, and fully comprehended, produces strange surprises and unexpected results. The majority of the readers of this article have doubtless often feasted on the tempting fruit of the honey-locust, which, when fully tinged by the autumn frosts, and still further shriveled up by the chilly blasts of early winter, affords a flavor both lucious and appetising to many palates. Uninviting in its appearance, and repelling with its prickly thorns, the honey-locust tree would scarcely have been selected as one possessed of remarkable remedial virtues. Such, however, is the function it seems now to be possessed of.

Through the discovery of Mr. Goodman, a veterinary surgeon of Louisiana, it was ascertained by a mere accident that the leaves of the tree were possessed of analgesic and anæsthetic properties of a most marked character. Mr. Goodman submitted the leaves of the tree to Dr. A. M. Seward, who isolated an alkaloid, to which he gave the name of *stenocarpine.* Stenocarpine was then submitted to Dr. J. Herbert Claiborne, Jr., and to Dr. Knapp, both of New York, who carefully studied its physiological effects, and communicated their results to the medical world. In an article in the *Medical Record* (October 1, 1887), Dr. Claiborne presents a very instructive account of the honey-locust tree from a botanical standpoint, and then gives the results of his experiments with the active leaves employed as an analgesic and anæsthetic. Dr. Claiborne has ascertained that five or six drops of a two per cent. solution of stenocarpine instilled into the eye produced complete anæsthesia of the conjunctiva and cornea within five minutes, and that anæsthesia lasted fully twenty minutes. He further observed that a one-fifth of one per cent. solution was sufficient to accomplish the same anæsthetic effect. The drug produces marked bleaching of the conjunctival vessels and complete dilation of the pupil. It was found to possess the anæsthetic properties of cocaine and the mydriatic effect of atropine. Its use relieved the pain of iritis and the photophobia of trachomatous pannus and phlyctenulæ in the most brilliant way.

Its anæsthetic effect upon the mucous membrane of the nose was equally well marked. Suffering with a severe attack of coryza, Dr. Claiborne painted the whole anterior nares on both sides with the solution. In ten minutes his breathing was free, and all sensibility to touch in the nose was removed. "The entire organ felt deadened—not unlike the sensation produced by frost-bite."

Stenocarpine is more actively toxic than cocaine. Five drops were taken and repeated in an hour by Dr. Claiborne. Two hours and a half after the first dose had been taken, his pulse had lowered eighteen beats. The heart recovered its normal beat and character in from four to six hours.

A slight sense of constriction in the soft palate, and a sense of well-being and surface-glow were experienced. Drowsiness came on and lasted several hours, and left with a dry throat and husky voice, which persisted two or three hours longer.

The effects of the drug upon the skin have not been fully proven. Dr. Claiborne asserts that it has an anæsthetizing effect on the unbroken, uninflamed cutis, whilst Dr. Knapp's observations fail to confirm this opinion. It is just here that the properties of cocaine and stenocarpine seem to differ. Cocaine has no influence upon a cutaneous surface, and should stenocarpine possess this latter property to the extent indicated by Dr. Claiborne, its value to surgery becomes immense.

The mydriatic effect of stenocarpine, in association with its anæsthetic properties, makes its use in ophthalmology of even greater value than cocaine. Whilst the two drugs have a number of properties in common, the differences in their physiological action favor a wider employment of local anæsthesia through their respective administration. It is probable that the discovery of stenocarpine will scarcely fall below that of cocaine itself in its beneficent results. A wider experience with the former agent is now needed to substantiate the full value of Mr. Goodman's discovery. Considering the wide distribution of the honey-locust tree and the abundance of its foliage, coupled with the remarkable strength of its alkaloid, the commercial status of the discovery is a most important one. Cocaine still remains too much of a luxury for universal use in surgical practice. A less expensive drug, with properties of equal and substantially the same value, is a great desideratum.—*Editorial Maryland Medical Journal.*

